# *rac*-*cis*,*cis*-Dicarbonyl­dichlorido{1-[2-(diphenyl­phosphan­yl)benz­yl]-3-mesityl­imidazol-2-yl­idene}ruthenium(II) dichloro­methane monosolvate

**DOI:** 10.1107/S1600536812032515

**Published:** 2012-07-25

**Authors:** Gregory J. Domski, Sallie A. Hohenboken, Dale C. Swenson

**Affiliations:** a Augustana College, Department of Chemistry, 639 38th Street, Rock Island, IL 61201, USA; b The University of Iowa, E331 Chemistry Building, Iowa City, IA 52242-1294, USA

## Abstract

The Ru^II^ atom in the title compound, [RuCl_2_(C_31_H_29_N_2_P)(CO)_2_]·CH_2_Cl_2_, exhibits a distorted octahedral coordination environment. The bond angles of the *cis* substituents at the Ru^II^ atom range from 82.72 (9) to 97.20 (3)°. This mol­ecule is of inter­est in the field of catalytic transfer hydrogenation.

## Related literature

For a review of transition metal catalysts supported by donor-functionalized *N*-heterocyclic carbenes (NHCs), see: Cavell & Normand (2008 ▶). For the first reported synthesis of the imidazolium chloride pro-ligand, see: Wang *et al.* (2005 ▶). For the structure of a similar compound incorporating an orthometalated *N*-phenyl group, see: Domski *et al.* (2012 ▶).
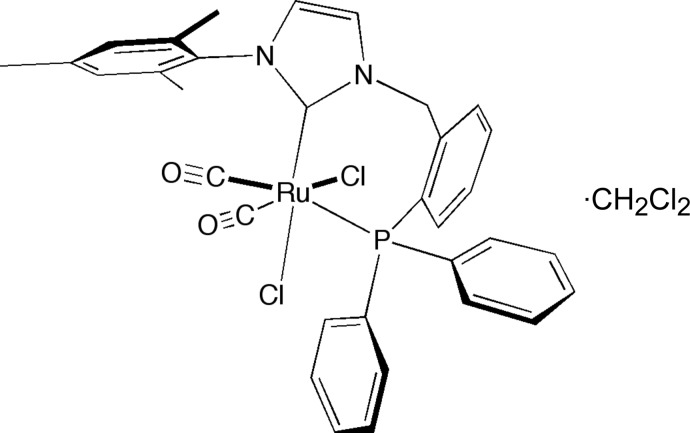


## Experimental

### 

#### Crystal data

[RuCl_2_(C_31_H_29_N_2_P)(CO)_2_]·CH_2_Cl_2_
*M* *_r_* = 773.45
Monoclinic, 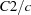

*a* = 22.539 (3) Å
*b* = 16.4065 (17) Å
*c* = 19.852 (2) Å
β = 111.004 (5)°
*V* = 6853.2 (13) Å^3^
*Z* = 8
Mo *K*α radiation
μ = 0.85 mm^−1^
*T* = 190 K
0.21 × 0.20 × 0.19 mm

#### Data collection

Nonius KappaCCD diffractometer
Absorption correction: multi-scan (*SCALEPACK*; Otwinowski & Minor, 1997 ▶) *T*_min_ = 0.842, *T*_max_ = 0.855
53042 measured reflections
7865 independent reflections
6109 reflections with *I* > 2σ(*I*)
*R*_int_ = 0.043

#### Refinement

*R*[*F* ^2^ > 2σ(*F* ^2^)] = 0.038
*wR*(*F* ^2^) = 0.097
*S* = 1.08
7865 reflections
402 parameters
H-atom parameters constrained
Δρ_max_ = 0.98 e Å^−3^
Δρ_min_ = −0.99 e Å^−3^

### 

Data collection: *COLLECT* (Nonius, 1997 ▶); cell refinement: *SCALEPACK* (Otwinowski & Minor, 1997 ▶); data reduction: *DENZO* (Otwinowski & Minor, 1997 ▶) and *SCALEPACK*; program(s) used to solve structure: *SHELXTL* (Sheldrick, 2008 ▶); program(s) used to refine structure: *SHELXTL*; molecular graphics: *SHELXTL*; software used to prepare material for publication: *SHELXTL*.

## Supplementary Material

Crystal structure: contains datablock(s) I, global. DOI: 10.1107/S1600536812032515/bt5975sup1.cif

Structure factors: contains datablock(s) I. DOI: 10.1107/S1600536812032515/bt5975Isup2.hkl

Additional supplementary materials:  crystallographic information; 3D view; checkCIF report

## Figures and Tables

**Fig. 1.**
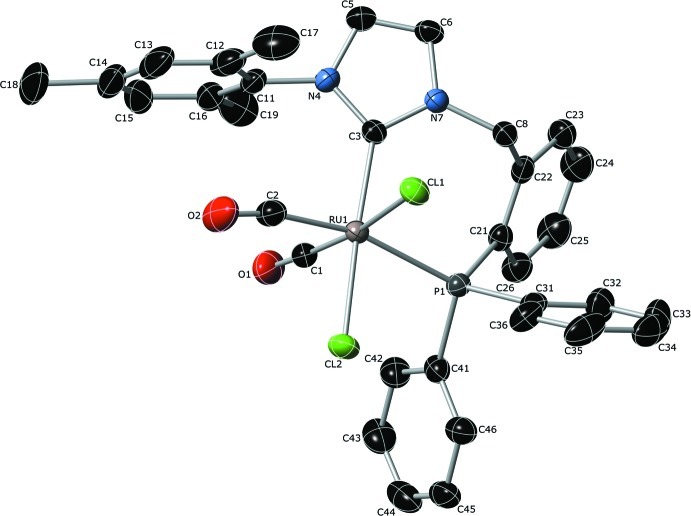
The molecular structure of the title complex with ellipsoids drawn at the 50% probability level. Hydrogen atoms and a dichloromethane molecule of crystallization were omitted for clarity.

## supplementary crystallographic information

### Comment

The title compound was prepared in order to prevent orthometalation which we had observed with a similar complex bearing an *N*-phenyl moiety (Domski *et al.*, 2012) with the ultimate goal of probing the effect of orthometalation on the catalytic behavior of ruthenium(II) complexes supported by phosphine-functionalized NHCs.

The complex exhibited a distorted octahedral geometry about ruthenium with a P1—Ru—Cl1 bond angle of 97.20 (3)°.

### Experimental

Single crystals suitable for X-ray diffraction studies were grown by vapor diffusion of diethyl ether onto a saturated dichloromethane solution of the title compound.

### Refinement

All H atoms were included with the riding model using the XL program default values. No further restraints or constraints were imposed on the refinement model.

### Crystal data

**Table:**

[RuCl_2_(C_31_H_29_N_2_P)(CO)_2_]·CH_2_Cl_2_	*F*(000) = 3136
*M**_r_* = 773.45	*D*_x_ = 1.499 Mg m^−^^3^
Monoclinic, *C*2/*c*	Mo *K*α radiation, λ = 0.71073 Å
Hall symbol: -C 2yc	Cell parameters from 15477 reflections
*a* = 22.539 (3) Å	θ = 1.0–27.9°
*b* = 16.4065 (17) Å	µ = 0.85 mm^−^^1^
*c* = 19.852 (2) Å	*T* = 190 K
β = 111.004 (5)°	Prism, yellow
*V* = 6853.2 (13) Å^3^	0.21 × 0.20 × 0.19 mm
*Z* = 8	

### Data collection

**Table:**

Nonius KappaCCD diffractometer	7865 independent reflections
Radiation source: fine-focus sealed tube	6109 reflections with *I* > 2σ(*I*)
Graphite monochromator	*R*_int_ = 0.043
Detector resolution: 9 pixels mm^-1^	θ_max_ = 27.5°, θ_min_ = 3.0°
CCD phi and ω scans	*h* = −29→29
Absorption correction: multi-scan (*SCALEPACK*; Otwinowski & Minor, 1997)	*k* = −20→21
*T*_min_ = 0.842, *T*_max_ = 0.855	*l* = −25→25
53042 measured reflections	

### Refinement

**Table:**

Refinement on *F*^2^	Primary atom site location: structure-invariant direct methods
Least-squares matrix: full	Secondary atom site location: difference Fourier map
*R*[*F*^2^ > 2σ(*F*^2^)] = 0.038	Hydrogen site location: inferred from neighbouring sites
*wR*(*F*^2^) = 0.097	H-atom parameters constrained
*S* = 1.08	*w* = 1/[σ^2^(*F*_o_^2^) + (0.0411*P*)^2^ + 13.2763*P*] where *P* = (*F*_o_^2^ + 2*F*_c_^2^)/3
7865 reflections	(Δ/σ)_max_ = 0.002
402 parameters	Δρ_max_ = 0.98 e Å^−^^3^
0 restraints	Δρ_min_ = −0.99 e Å^−^^3^

### Special details

**Table:**

Experimental. In a nitrogen-filled glove box, a Schlenk flask was charged with 1-mesityl-3-(2-diphenylphosphinobenzyl)-1*H*-imidazol-3-ium chloride (0.7625 g), Ag_2_O (0.3659 g), and 4 Å molecular seives (*ca* 0.5 g). The solids were suspended in dry, degassed dichloromethane and allowed to stir overnight in the dark. After 24 h, the reaction mixture was filtered through Celite^TM^ into a Schlenk flask that had been charged with [Ru(CO)_3_Cl_2_]_2_ (0.4070 g) under a nitrogen atmoshphere. The reaction mixture was allowed to stir at room temperature in the dark overnight. After 24 h, the reaction mixture was filtered through Celite^TM^ and the volatiles were removed *in vacuo* to furnish a yellow solid. The crude product was purified by column chromatography (SiO_2_, 40:1 CH_2_Cl_2_/MeOH). Single crystals of the title compound were obtained by slow diffusion of diethyl ether onto a concentrated solution of the yellow powder isolated *via* column chromatography in dichloromethane.
Geometry. All e.s.d.'s (except the e.s.d. in the dihedral angle between two l.s. planes) are estimated using the full covariance matrix. The cell e.s.d.'s are taken into account individually in the estimation of e.s.d.'s in distances, angles and torsion angles; correlations between e.s.d.'s in cell parameters are only used when they are defined by crystal symmetry. An approximate (isotropic) treatment of cell e.s.d.'s is used for estimating e.s.d.'s involving l.s. planes.
Refinement. Refinement of *F*^2^ against ALL reflections. The weighted *R*-factor *wR* and goodness of fit *S* are based on *F*^2^, conventional *R*-factors *R* are based on *F*, with *F* set to zero for negative *F*^2^. The threshold expression of *F*^2^ > σ(*F*^2^) is used only for calculating *R*-factors(gt) *etc*. and is not relevant to the choice of reflections for refinement. *R*-factors based on *F*^2^ are statistically about twice as large as those based on *F*, and *R*- factors based on ALL data will be even larger.Several low angle reflections were omitted from the final cycles of refinement due to beam-stop beam shadowing effects.

### Fractional atomic coordinates and isotropic or equivalent isotropic displacement parameters (Å^2^)

**Table:**

	*x*	*y*	*z*	*U*_iso_*/*U*_eq_	
Ru1	0.703359 (10)	0.039133 (12)	0.587686 (11)	0.02261 (7)	
Cl1	0.66435 (3)	0.17943 (4)	0.58161 (4)	0.03120 (16)	
Cl2	0.68354 (4)	0.02104 (5)	0.70046 (4)	0.03544 (17)	
C1	0.72360 (14)	−0.06922 (18)	0.58206 (15)	0.0308 (6)	
O1	0.73228 (12)	−0.13645 (13)	0.57587 (12)	0.0466 (6)	
C2	0.61471 (14)	0.00921 (19)	0.54483 (16)	0.0337 (6)	
O2	0.56467 (11)	−0.01302 (18)	0.53158 (14)	0.0582 (7)	
C3	0.71699 (12)	0.06780 (15)	0.49266 (14)	0.0233 (5)	
N4	0.68600 (11)	0.03936 (13)	0.42422 (12)	0.0266 (5)	
C5	0.70667 (14)	0.07903 (18)	0.37477 (16)	0.0327 (6)	
H5	0.6921	0.0699	0.3242	0.039*	
C6	0.75093 (14)	0.13249 (17)	0.41214 (15)	0.0298 (6)	
H6	0.7740	0.1685	0.3932	0.036*	
N7	0.75680 (10)	0.12545 (13)	0.48352 (12)	0.0242 (5)	
C8	0.80416 (13)	0.17248 (16)	0.54067 (15)	0.0275 (6)	
H8A	0.7871	0.1858	0.5789	0.033*	
H8B	0.8129	0.2243	0.5204	0.033*	
C11	0.63333 (14)	−0.01646 (19)	0.40064 (15)	0.0330 (6)	
C12	0.57187 (16)	0.0169 (2)	0.38096 (17)	0.0465 (8)	
C13	0.52078 (18)	−0.0363 (3)	0.35720 (19)	0.0618 (12)	
H13	0.4788	−0.0153	0.3438	0.074*	
C14	0.5295 (2)	−0.1188 (3)	0.3527 (2)	0.0658 (13)	
C15	0.5905 (2)	−0.1488 (2)	0.36929 (19)	0.0598 (11)	
H15	0.5962	−0.2056	0.3645	0.072*	
C16	0.64434 (17)	−0.0981 (2)	0.39303 (17)	0.0418 (8)	
C17	0.56132 (18)	0.1068 (3)	0.3862 (2)	0.0619 (11)	
H17A	0.5163	0.1169	0.3776	0.093*	
H17B	0.5735	0.1358	0.3500	0.093*	
H17C	0.5873	0.1262	0.4345	0.093*	
C18	0.4727 (2)	−0.1763 (4)	0.3289 (2)	0.103 (2)	
H18A	0.4437	−0.1607	0.2806	0.154*	
H18B	0.4503	−0.1729	0.3630	0.154*	
H18C	0.4874	−0.2323	0.3276	0.154*	
C19	0.7094 (2)	−0.1301 (2)	0.4042 (2)	0.0580 (10)	
H19A	0.7409	−0.1004	0.4438	0.087*	
H19B	0.7186	−0.1226	0.3599	0.087*	
H19C	0.7114	−0.1882	0.4161	0.087*	
P1	0.81175 (3)	0.06515 (4)	0.66858 (4)	0.02367 (15)	
C21	0.87363 (12)	0.06917 (17)	0.62890 (15)	0.0266 (6)	
C22	0.86519 (13)	0.12488 (17)	0.57297 (15)	0.0280 (6)	
C23	0.91411 (14)	0.1368 (2)	0.54681 (17)	0.0372 (7)	
H23	0.9092	0.1761	0.5101	0.045*	
C25	0.97763 (15)	0.0365 (2)	0.62788 (19)	0.0442 (8)	
H25	1.0156	0.0054	0.6460	0.053*	
C24	0.96986 (15)	0.0922 (2)	0.57349 (19)	0.0469 (8)	
H24	1.0025	0.1000	0.5544	0.056*	
C26	0.93032 (14)	0.02579 (19)	0.65620 (17)	0.0358 (7)	
H26	0.9366	−0.0116	0.6947	0.043*	
C31	0.82793 (14)	0.16048 (16)	0.72018 (14)	0.0280 (6)	
C32	0.88838 (17)	0.1950 (2)	0.74234 (18)	0.0454 (8)	
H32	0.9211	0.1686	0.7309	0.055*	
C33	0.9013 (2)	0.2668 (2)	0.7806 (2)	0.0579 (10)	
H33	0.9428	0.2896	0.7958	0.069*	
C34	0.8540 (2)	0.3057 (2)	0.79685 (19)	0.0555 (10)	
H34	0.8626	0.3559	0.8224	0.067*	
C35	0.79473 (19)	0.2724 (2)	0.77632 (18)	0.0523 (9)	
H35	0.7624	0.2993	0.7881	0.063*	
C36	0.78110 (16)	0.19921 (19)	0.73826 (16)	0.0388 (7)	
H36	0.7399	0.1760	0.7248	0.047*	
C41	0.83938 (13)	−0.01377 (17)	0.73767 (16)	0.0294 (6)	
C42	0.85110 (14)	−0.09220 (18)	0.71899 (18)	0.0381 (7)	
H42	0.8445	−0.1047	0.6701	0.046*	
C43	0.87236 (15)	−0.1522 (2)	0.7712 (2)	0.0453 (8)	
H43	0.8802	−0.2057	0.7581	0.054*	
C44	0.88213 (15)	−0.1345 (2)	0.8424 (2)	0.0479 (9)	
H44	0.8973	−0.1756	0.8782	0.057*	
C45	0.87005 (15)	−0.0579 (2)	0.86169 (18)	0.0440 (8)	
H45	0.8765	−0.0460	0.9107	0.053*	
C46	0.84819 (14)	0.0031 (2)	0.80915 (17)	0.0352 (7)	
H46	0.8394	0.0560	0.8225	0.042*	
C51	0.8875 (3)	−0.2190 (4)	0.4700 (3)	0.1036 (19)	
H51B	0.8585	−0.2558	0.4830	0.124*	
H51A	0.8671	−0.2054	0.4182	0.124*	
Cl12	0.89756 (8)	−0.12965 (10)	0.52043 (9)	0.1097 (5)	
Cl11	0.95810 (9)	−0.27001 (8)	0.48306 (7)	0.0993 (5)	

### Atomic displacement parameters (Å^2^)

**Table:**

	*U*^11^	*U*^22^	*U*^33^	*U*^12^	*U*^13^	*U*^23^
Ru1	0.02264 (11)	0.02248 (11)	0.02245 (12)	−0.00064 (8)	0.00778 (9)	0.00257 (8)
Cl1	0.0356 (4)	0.0259 (3)	0.0350 (4)	0.0041 (3)	0.0161 (3)	0.0041 (3)
Cl2	0.0377 (4)	0.0426 (4)	0.0292 (4)	−0.0005 (3)	0.0158 (3)	0.0074 (3)
C1	0.0334 (15)	0.0323 (16)	0.0241 (15)	−0.0065 (12)	0.0069 (12)	0.0018 (12)
O1	0.0576 (15)	0.0300 (12)	0.0451 (14)	0.0001 (10)	0.0099 (12)	−0.0012 (10)
C2	0.0309 (16)	0.0417 (17)	0.0277 (16)	−0.0002 (13)	0.0094 (13)	0.0029 (13)
O2	0.0288 (13)	0.096 (2)	0.0461 (15)	−0.0127 (13)	0.0087 (11)	0.0031 (14)
C3	0.0234 (13)	0.0207 (12)	0.0259 (14)	0.0016 (10)	0.0089 (11)	0.0030 (10)
N4	0.0280 (12)	0.0290 (12)	0.0216 (12)	−0.0029 (10)	0.0074 (10)	−0.0017 (9)
C5	0.0377 (16)	0.0375 (16)	0.0241 (15)	−0.0016 (13)	0.0125 (13)	0.0006 (12)
C6	0.0351 (15)	0.0328 (15)	0.0253 (14)	−0.0002 (12)	0.0156 (12)	0.0035 (12)
N7	0.0271 (11)	0.0227 (11)	0.0233 (12)	−0.0012 (9)	0.0094 (9)	0.0001 (9)
C8	0.0311 (14)	0.0266 (13)	0.0260 (14)	−0.0046 (11)	0.0119 (12)	−0.0007 (11)
C11	0.0314 (15)	0.0433 (17)	0.0218 (14)	−0.0114 (13)	0.0066 (12)	−0.0024 (12)
C12	0.0356 (17)	0.074 (2)	0.0263 (16)	−0.0062 (17)	0.0067 (14)	0.0025 (16)
C13	0.0363 (19)	0.111 (4)	0.0307 (19)	−0.022 (2)	0.0034 (15)	−0.002 (2)
C14	0.059 (3)	0.106 (4)	0.0304 (19)	−0.047 (3)	0.0138 (18)	−0.014 (2)
C15	0.092 (3)	0.052 (2)	0.040 (2)	−0.037 (2)	0.028 (2)	−0.0159 (17)
C16	0.057 (2)	0.0421 (18)	0.0282 (16)	−0.0172 (16)	0.0176 (15)	−0.0090 (13)
C17	0.049 (2)	0.078 (3)	0.053 (2)	0.025 (2)	0.0124 (19)	0.018 (2)
C18	0.090 (4)	0.160 (5)	0.053 (3)	−0.092 (4)	0.019 (3)	−0.020 (3)
C19	0.084 (3)	0.0394 (19)	0.061 (2)	0.0014 (19)	0.038 (2)	−0.0089 (17)
P1	0.0234 (3)	0.0244 (3)	0.0224 (3)	0.0005 (3)	0.0072 (3)	0.0008 (3)
C21	0.0225 (13)	0.0288 (13)	0.0284 (14)	−0.0017 (11)	0.0091 (11)	−0.0027 (11)
C22	0.0267 (14)	0.0310 (14)	0.0264 (14)	−0.0073 (11)	0.0095 (12)	−0.0070 (11)
C23	0.0326 (16)	0.0475 (18)	0.0337 (17)	−0.0091 (13)	0.0144 (14)	−0.0021 (14)
C25	0.0243 (15)	0.059 (2)	0.048 (2)	0.0048 (14)	0.0111 (14)	−0.0013 (16)
C24	0.0290 (16)	0.069 (2)	0.048 (2)	−0.0061 (16)	0.0198 (15)	−0.0029 (18)
C26	0.0287 (15)	0.0429 (17)	0.0345 (17)	0.0016 (13)	0.0098 (13)	0.0002 (13)
C31	0.0356 (15)	0.0262 (14)	0.0198 (13)	−0.0006 (11)	0.0071 (12)	0.0006 (11)
C32	0.049 (2)	0.0439 (19)	0.044 (2)	−0.0121 (16)	0.0175 (16)	−0.0131 (15)
C33	0.070 (3)	0.052 (2)	0.052 (2)	−0.030 (2)	0.022 (2)	−0.0216 (18)
C34	0.089 (3)	0.0314 (17)	0.0350 (19)	−0.0025 (18)	0.008 (2)	−0.0074 (14)
C35	0.066 (2)	0.048 (2)	0.0330 (18)	0.0184 (18)	0.0063 (17)	−0.0109 (15)
C36	0.0393 (17)	0.0433 (18)	0.0292 (16)	0.0057 (14)	0.0067 (14)	−0.0062 (13)
C41	0.0200 (13)	0.0308 (14)	0.0340 (16)	0.0006 (11)	0.0056 (12)	0.0060 (12)
C42	0.0335 (16)	0.0335 (16)	0.0408 (18)	0.0033 (13)	0.0056 (14)	0.0076 (13)
C43	0.0368 (18)	0.0343 (17)	0.057 (2)	0.0075 (14)	0.0080 (16)	0.0144 (15)
C44	0.0339 (17)	0.050 (2)	0.057 (2)	0.0072 (15)	0.0137 (16)	0.0301 (17)
C45	0.0353 (17)	0.060 (2)	0.0353 (18)	−0.0006 (15)	0.0111 (14)	0.0189 (16)
C46	0.0306 (15)	0.0409 (17)	0.0345 (17)	−0.0001 (13)	0.0121 (13)	0.0073 (13)
C51	0.110 (5)	0.108 (4)	0.095 (4)	−0.045 (4)	0.038 (4)	−0.044 (3)
Cl12	0.1207 (12)	0.1043 (11)	0.1156 (12)	−0.0406 (9)	0.0562 (10)	−0.0495 (9)
Cl11	0.1521 (14)	0.0775 (8)	0.0702 (8)	−0.0089 (9)	0.0423 (9)	0.0073 (6)

### Geometric parameters (Å, º)

**Table:**

Ru1—C1	1.849 (3)	P1—C41	1.826 (3)
Ru1—C2	1.934 (3)	P1—C21	1.832 (3)
Ru1—C3	2.071 (3)	P1—C31	1.833 (3)
Ru1—P1	2.4325 (8)	C21—C26	1.392 (4)
Ru1—Cl1	2.4515 (7)	C21—C22	1.398 (4)
Ru1—Cl2	2.4529 (8)	C22—C23	1.391 (4)
C1—O1	1.135 (4)	C23—C24	1.384 (5)
C2—O2	1.123 (4)	C23—H23	0.9500
C3—N7	1.360 (3)	C25—C24	1.377 (5)
C3—N4	1.368 (3)	C25—C26	1.383 (4)
N4—C5	1.390 (4)	C25—H25	0.9500
N4—C11	1.438 (4)	C24—H24	0.9500
C5—C6	1.336 (4)	C26—H26	0.9500
C5—H5	0.9500	C31—C36	1.385 (4)
C6—N7	1.380 (3)	C31—C32	1.393 (4)
C6—H6	0.9500	C32—C33	1.376 (5)
N7—C8	1.467 (3)	C32—H32	0.9500
C8—C22	1.510 (4)	C33—C34	1.376 (6)
C8—H8A	0.9900	C33—H33	0.9500
C8—H8B	0.9900	C34—C35	1.363 (6)
C11—C16	1.380 (5)	C34—H34	0.9500
C11—C12	1.408 (5)	C35—C36	1.393 (4)
C12—C13	1.386 (5)	C35—H35	0.9500
C12—C17	1.503 (5)	C36—H36	0.9500
C13—C14	1.376 (6)	C41—C46	1.388 (4)
C13—H13	0.9500	C41—C42	1.390 (4)
C14—C15	1.385 (6)	C42—C43	1.385 (4)
C14—C18	1.522 (5)	C42—H42	0.9500
C15—C16	1.407 (5)	C43—C44	1.381 (5)
C15—H15	0.9500	C43—H43	0.9500
C16—C19	1.498 (5)	C44—C45	1.369 (5)
C17—H17A	0.9800	C44—H44	0.9500
C17—H17B	0.9800	C45—C46	1.401 (4)
C17—H17C	0.9800	C45—H45	0.9500
C18—H18A	0.9800	C46—H46	0.9500
C18—H18B	0.9800	C51—Cl11	1.733 (6)
C18—H18C	0.9800	C51—Cl12	1.744 (5)
C19—H19A	0.9800	C51—H51B	0.9900
C19—H19B	0.9800	C51—H51A	0.9900
C19—H19C	0.9800		
			
C1—Ru1—C2	88.15 (13)	H19A—C19—H19B	109.5
C1—Ru1—C3	92.46 (11)	C16—C19—H19C	109.5
C2—Ru1—C3	97.13 (11)	H19A—C19—H19C	109.5
C1—Ru1—P1	89.86 (9)	H19B—C19—H19C	109.5
C2—Ru1—P1	165.93 (9)	C41—P1—C21	103.96 (13)
C3—Ru1—P1	96.87 (7)	C41—P1—C31	103.84 (13)
C1—Ru1—Cl1	172.69 (9)	C21—P1—C31	100.37 (13)
C2—Ru1—Cl1	85.46 (9)	C41—P1—Ru1	111.28 (9)
C3—Ru1—Cl1	84.81 (7)	C21—P1—Ru1	117.49 (9)
P1—Ru1—Cl1	97.20 (3)	C31—P1—Ru1	118.04 (9)
C1—Ru1—Cl2	93.92 (9)	C26—C21—C22	119.2 (3)
C2—Ru1—Cl2	82.72 (9)	C26—C21—P1	123.3 (2)
C3—Ru1—Cl2	173.60 (7)	C22—C21—P1	117.2 (2)
P1—Ru1—Cl2	83.52 (3)	C23—C22—C21	119.2 (3)
Cl1—Ru1—Cl2	88.80 (3)	C23—C22—C8	119.5 (3)
O1—C1—Ru1	175.8 (3)	C21—C22—C8	121.3 (2)
O2—C2—Ru1	168.0 (3)	C24—C23—C22	121.0 (3)
N7—C3—N4	103.4 (2)	C24—C23—H23	119.5
N7—C3—Ru1	126.86 (19)	C22—C23—H23	119.5
N4—C3—Ru1	129.61 (19)	C24—C25—C26	120.2 (3)
C3—N4—C5	111.1 (2)	C24—C25—H25	119.9
C3—N4—C11	127.5 (2)	C26—C25—H25	119.9
C5—N4—C11	121.0 (2)	C25—C24—C23	119.6 (3)
C6—C5—N4	106.7 (2)	C25—C24—H24	120.2
C6—C5—H5	126.6	C23—C24—H24	120.2
N4—C5—H5	126.6	C25—C26—C21	120.7 (3)
C5—C6—N7	107.0 (2)	C25—C26—H26	119.7
C5—C6—H6	126.5	C21—C26—H26	119.7
N7—C6—H6	126.5	C36—C31—C32	118.8 (3)
C3—N7—C6	111.7 (2)	C36—C31—P1	121.3 (2)
C3—N7—C8	126.5 (2)	C32—C31—P1	120.0 (2)
C6—N7—C8	121.7 (2)	C33—C32—C31	120.8 (3)
N7—C8—C22	110.8 (2)	C33—C32—H32	119.6
N7—C8—H8A	109.5	C31—C32—H32	119.6
C22—C8—H8A	109.5	C32—C33—C34	119.9 (4)
N7—C8—H8B	109.5	C32—C33—H33	120.1
C22—C8—H8B	109.5	C34—C33—H33	120.1
H8A—C8—H8B	108.1	C35—C34—C33	120.1 (3)
C16—C11—C12	123.0 (3)	C35—C34—H34	119.9
C16—C11—N4	119.6 (3)	C33—C34—H34	119.9
C12—C11—N4	117.2 (3)	C34—C35—C36	120.7 (3)
C13—C12—C11	117.7 (4)	C34—C35—H35	119.7
C13—C12—C17	120.6 (4)	C36—C35—H35	119.7
C11—C12—C17	121.8 (3)	C31—C36—C35	119.7 (3)
C14—C13—C12	121.4 (4)	C31—C36—H36	120.1
C14—C13—H13	119.3	C35—C36—H36	120.1
C12—C13—H13	119.3	C46—C41—C42	119.0 (3)
C13—C14—C15	119.1 (3)	C46—C41—P1	120.8 (2)
C13—C14—C18	120.4 (5)	C42—C41—P1	120.2 (2)
C15—C14—C18	120.4 (5)	C43—C42—C41	120.4 (3)
C14—C15—C16	122.2 (4)	C43—C42—H42	119.8
C14—C15—H15	118.9	C41—C42—H42	119.8
C16—C15—H15	118.9	C44—C43—C42	120.1 (3)
C11—C16—C15	116.4 (3)	C44—C43—H43	119.9
C11—C16—C19	121.9 (3)	C42—C43—H43	119.9
C15—C16—C19	121.5 (3)	C45—C44—C43	120.3 (3)
C12—C17—H17A	109.5	C45—C44—H44	119.9
C12—C17—H17B	109.5	C43—C44—H44	119.9
H17A—C17—H17B	109.5	C44—C45—C46	120.0 (3)
C12—C17—H17C	109.5	C44—C45—H45	120.0
H17A—C17—H17C	109.5	C46—C45—H45	120.0
H17B—C17—H17C	109.5	C41—C46—C45	120.2 (3)
C14—C18—H18A	109.5	C41—C46—H46	119.9
C14—C18—H18B	109.5	C45—C46—H46	119.9
H18A—C18—H18B	109.5	Cl11—C51—Cl12	113.4 (3)
C14—C18—H18C	109.5	Cl11—C51—H51B	108.9
H18A—C18—H18C	109.5	Cl12—C51—H51B	108.9
H18B—C18—H18C	109.5	Cl11—C51—H51A	108.9
C16—C19—H19A	109.5	Cl12—C51—H51A	108.9
C16—C19—H19B	109.5	H51B—C51—H51A	107.7
			
C1—Ru1—C2—O2	77.2 (14)	C3—Ru1—P1—C21	−17.66 (12)
C3—Ru1—C2—O2	169.5 (14)	Cl1—Ru1—P1—C21	−103.27 (10)
P1—Ru1—C2—O2	−4.8 (18)	Cl2—Ru1—P1—C21	168.76 (10)
Cl1—Ru1—C2—O2	−106.3 (14)	C1—Ru1—P1—C31	−164.73 (13)
Cl2—Ru1—C2—O2	−17.0 (14)	C2—Ru1—P1—C31	−82.9 (4)
C1—Ru1—C3—N7	−123.9 (2)	C3—Ru1—P1—C31	102.81 (12)
C2—Ru1—C3—N7	147.6 (2)	Cl1—Ru1—P1—C31	17.20 (10)
P1—Ru1—C3—N7	−33.8 (2)	Cl2—Ru1—P1—C31	−70.76 (10)
Cl1—Ru1—C3—N7	62.9 (2)	C41—P1—C21—C26	−6.8 (3)
C1—Ru1—C3—N4	61.6 (2)	C31—P1—C21—C26	100.4 (3)
C2—Ru1—C3—N4	−26.9 (3)	Ru1—P1—C21—C26	−130.2 (2)
P1—Ru1—C3—N4	151.7 (2)	C41—P1—C21—C22	179.6 (2)
Cl1—Ru1—C3—N4	−111.6 (2)	C31—P1—C21—C22	−73.2 (2)
N7—C3—N4—C5	0.0 (3)	Ru1—P1—C21—C22	56.2 (2)
Ru1—C3—N4—C5	175.5 (2)	C26—C21—C22—C23	−1.2 (4)
N7—C3—N4—C11	−172.7 (3)	P1—C21—C22—C23	172.7 (2)
Ru1—C3—N4—C11	2.7 (4)	C26—C21—C22—C8	179.0 (3)
C3—N4—C5—C6	0.1 (3)	P1—C21—C22—C8	−7.1 (3)
C11—N4—C5—C6	173.4 (3)	N7—C8—C22—C23	94.5 (3)
N4—C5—C6—N7	−0.2 (3)	N7—C8—C22—C21	−85.7 (3)
N4—C3—N7—C6	−0.1 (3)	C21—C22—C23—C24	2.4 (4)
Ru1—C3—N7—C6	−175.79 (19)	C8—C22—C23—C24	−177.8 (3)
N4—C3—N7—C8	−176.3 (2)	C26—C25—C24—C23	−0.7 (5)
Ru1—C3—N7—C8	8.1 (4)	C22—C23—C24—C25	−1.4 (5)
C5—C6—N7—C3	0.2 (3)	C24—C25—C26—C21	1.9 (5)
C5—C6—N7—C8	176.6 (2)	C22—C21—C26—C25	−0.9 (4)
C3—N7—C8—C22	83.6 (3)	P1—C21—C26—C25	−174.4 (2)
C6—N7—C8—C22	−92.2 (3)	C41—P1—C31—C36	−96.4 (3)
C3—N4—C11—C16	−97.6 (3)	C21—P1—C31—C36	156.2 (2)
C5—N4—C11—C16	90.3 (3)	Ru1—P1—C31—C36	27.3 (3)
C3—N4—C11—C12	87.2 (4)	C41—P1—C31—C32	83.2 (3)
C5—N4—C11—C12	−84.9 (3)	C21—P1—C31—C32	−24.1 (3)
C16—C11—C12—C13	4.1 (5)	Ru1—P1—C31—C32	−153.1 (2)
N4—C11—C12—C13	179.1 (3)	C36—C31—C32—C33	−0.9 (5)
C16—C11—C12—C17	−176.7 (3)	P1—C31—C32—C33	179.5 (3)
N4—C11—C12—C17	−1.7 (4)	C31—C32—C33—C34	−0.6 (6)
C11—C12—C13—C14	−0.2 (5)	C32—C33—C34—C35	1.3 (6)
C17—C12—C13—C14	−179.4 (4)	C33—C34—C35—C36	−0.6 (6)
C12—C13—C14—C15	−2.8 (6)	C32—C31—C36—C35	1.6 (5)
C12—C13—C14—C18	177.9 (3)	P1—C31—C36—C35	−178.8 (2)
C13—C14—C15—C16	2.1 (6)	C34—C35—C36—C31	−0.9 (5)
C18—C14—C15—C16	−178.6 (3)	C21—P1—C41—C46	123.2 (2)
C12—C11—C16—C15	−4.7 (5)	C31—P1—C41—C46	18.6 (3)
N4—C11—C16—C15	−179.6 (3)	Ru1—P1—C41—C46	−109.4 (2)
C12—C11—C16—C19	171.0 (3)	C21—P1—C41—C42	−57.6 (3)
N4—C11—C16—C19	−3.9 (4)	C31—P1—C41—C42	−162.3 (2)
C14—C15—C16—C11	1.5 (5)	Ru1—P1—C41—C42	69.8 (2)
C14—C15—C16—C19	−174.2 (3)	C46—C41—C42—C43	−1.2 (4)
C1—Ru1—P1—C41	−44.83 (14)	P1—C41—C42—C43	179.7 (2)
C2—Ru1—P1—C41	37.0 (4)	C41—C42—C43—C44	−0.1 (5)
C3—Ru1—P1—C41	−137.30 (13)	C42—C43—C44—C45	0.9 (5)
Cl1—Ru1—P1—C41	137.09 (11)	C43—C44—C45—C46	−0.5 (5)
Cl2—Ru1—P1—C41	49.13 (11)	C42—C41—C46—C45	1.5 (4)
C1—Ru1—P1—C21	74.80 (14)	P1—C41—C46—C45	−179.3 (2)
C2—Ru1—P1—C21	156.6 (4)	C44—C45—C46—C41	−0.7 (5)
